# Low rate of seroconversion analyzing QuantiFERON TB-gold testing in patients with psoriasis and hidradenitis suppurativa taking biologic therapy at an academic center in New York City

**DOI:** 10.1016/j.jdin.2024.04.004

**Published:** 2024-05-10

**Authors:** Apostolos Katsiaunis, Jade Conway, Debra D'Angelo, Shari R. Lipner

**Affiliations:** a Tufts University School of Medicine, Boston, Massachusetts; b School of Medicine, New York Medical College, Valhalla, New York; c Division of Biostatistics, Department of Population Health Sciences, Weill Cornell Medicine, New York, New York; d Department of Dermatology, Weill Cornell Medicine, New York, New York

**Keywords:** biologics, hidradenitis suppurativa, psoriasis, QuantiFERON-TB gold, seroconversion

*To the Editor:* Current guidelines advise tuberculosis (TB) baseline screening before initiating biologic medications, due to risk of latent tuberculosis infection reactivation, with varying recommendations on repeat screening. Studies analyzing screening in US cities with high TB rates are lacking. We aimed to stratify utility of serial QuantiFERON TB-gold (QFT) testing in patients with psoriasis or hidradenitis taking biologic treatment for hidradenitis suppurativa in New York City (NYC), which has high TB burden.

Weill Cornell Medical Center’s EPIC database was queried March 23, 2011 to July 21, 2023 for psoriasis or patients with hidradenitis suppurativa with ages ≥18 years treated with a biologic, with ≥2 QFT test results, with ≥1 prior to treatment. Wilcoxon rank-sum tests evaluated mean ages and number of risk factors between seroconverters and nonseroconverters (*P* < .05). Receiver operating characteristic analysis with Youden index identified optimal age cutoff for seroconversion risk.

A total of 356 patients were included (Table I). Overall, 71.6% of patients resided in NYC, (TB burden 6.1 cases/100,000 people), and 90.1% lived within the New York-Newark-Jersey City metropolitan statistical area (top 10% for TB burden among all metropolitan statistical area’s with >500,000 residents in 2021, 4.2/100,000).^1^ Patients underwent mean 3 QFT tests (range: 2-12) (Supplementary Table I, available via Mendeley at https://data.mendeley.com/datasets/t4mj454jwy/1). Five patients seroconverted (1.4%), and 2 had negative repeat testing and were considered false positives on their second and fifth tests, respectively (0.6%). There were 3 true seroconverters (0.8%), all seroconverting on their second test. True seroconverters were older, on average, than other subjects (73 and 45.7 years, *P* < .008) and had greater numbers of TB risk factors (4.67 and 1.86, *P* = .0178) (Supplementary Table II, available via Mendeley at https://data.mendeley.com/datasets/t4mj454jwy/1). Sixty-six years old was the cutoff for increased seroconversion risk (receiver operating characteristic analysis, Youden). No seroconverters resided in high-TB risk neighborhoods (Table II). All 3 true seroconverters were treated for latent tuberculosis infection and none developed active TB.

**Table I:** Patient demographics, treatment details, and QuantiFERON TB-gold testing outcomes

Demographic characteristic	*n* (%)
Number of patients	356
Age (y), mean (Q1-Q3)	46.0 (32.0-59.0)
Sex, *n* (%)	
Male	164 (46.1)
Female	192 (53.9)
Race, *n* (%)	
White	138 (38.8)
Other	97 (27.2)
Declined	56 (15.7)
Black or African American	46 (12.9)
Asian	19 (5.3)
Ethnicity, *n* (%)	
Not Hispanic, Latino, or Spanish origin	170 (47.8)
Hispanic, Latino, or Spanish origin	101 (28.4)
Declined	85 (23.9)
Geographic distribution by state, *n* (%)	
NY	301 (84.5)
NJ	41 (11.5)
Others∗	14 (3.9)
Geographic distribution within NY, *n* (%)	
Manhattan	113 (31.7)
Queens	53 (14.9)
Bronx	45 (12.6)
Brooklyn	41 (11.5)
Staten Island	3 (0.8)
Total within New York City (NYC)	255 (71.6)
High tuberculosis risk zip code†	18 (5.1)
Treatment, *n* (%)	
Adalimumab	120 (33.7)
Ustekinumab	61 (17.1)
Secukinumab	48 (13.5)
Risankizumab	42 (11.8)
Infliximab	33 (9.3)
Etanercept	20 (5.6)
Guselkumab	17 (4.8)
Ixekizumab	15 (4.2)
Abatacept	3 (0.8)
Certolizumab	3 (0.8)
Tildrakizumab	2 (0.6)
Vedolizumab	1 (0.3)
Dupilumab	1 (0.3)
Indication, *n* (%)	
Psoriasis	321 (90.2)
Hidradenitis suppurativa	35 (9.8)
Number of tests per patient, median (Q1-Q3)	3 (2-4)
Time between first and last test (mo), median (Q1-Q3)	35 (22-51)
Final outcomes, *n* (%)	
Persistently seronegative	336 (94.4)
Persistently seropositive	6 (1.7)
Seroreversion	5 (1.4)
Seroconversion	3 (0.8)
False seroconversion	2 (0.6)
Others	4 (1.1)

∗ Connecticut, Pennsylvania, Texas, North Carolina, Virginia, Florida, Wisconsin, Delaware, New York, New Jersey.

† Includes ZIP codes within NYC at or above 10 active tuberculosis cases/100,000 people.

**Table II:** Demographics and outcomes of patients who seroconverted

#	Sex	Age (y)	Zip code TB risk level∗	Race and ethnicity	Treatment	Risk factors	Duration of therapy prior to conversion (mo)	Further management
1	M	32	Low	White, not Hispanic, Latino, or Spanish origin	Adalimumab	Opioid use disorder, IV drug use, exposure to tuberculosis through close contact, methotrexate use	20	Patient reported night sweats. Repeat QuantiFERON Gold test, AFB culture, CXR, and PPD were negative, no treatment for LTBI (FC)
2	M	78	Low	White, declined	Ustekinumab	Smoking, T2DM	12	Treated with rifampin for 2 mo, discontinued due to abnormal LFT's
3	M	71	Low	Declined	Adalimumab, guselkumab	None known	11 (adalimumab) 1 (guselkumab)	Repeat QuantiFERON Gold test was negative, patient continued guselkumab, no treatment for LTBI (FC)
4	F	66	Low	White, not Hispanic, Latino, or Spanish origin	Etanercept, ustekinumab	Crohn's disease, smoking	24	During etanercept treatment had positive QuantiFERON-TB Gold Plus, followed by normal CXR and 9 mo of INH. Four y after that, had 2 subsequent tests that were negative and positive, followed by 2 y of ustekinumab.
5	M	75	Low	Other, not Hispanic, Latino, or Spanish origin	Secukinumab	Positive PPD's 50 y ago, smoking, T2DM, hepatitis B, internal medicine physician with regular contact with immigrants as patient population	6	Treated with 9 mo of INH

*AFB*, Acid-fast bacillus; *CXR*, chest x-ray; *FC*, False conversion; *INH*, isoniazid; *IV*, intravenous; *LTBI*, latent tuberculosis infection; *PPD*, purified protein derivative; *TB*, tuberculosis.

∗ Low zip code tuberculosis risk level indicates under 10 active tuberculosis cases per 100,000 people.

We found a low rate of seroconversion (<1%) in patient taking biologics for dermatologic conditions in NYC, which has relatively high TB prevalence. Seroconversion rates in our study were surprisingly similar to studies conducted in cities with lower TB burdens. A retrospective study of 570 psoriasis patients taking TNF alpha inhibitors (TNFIs) in Iowa City, Iowa (TB burden 1.5/100,000), reported only 1 (0.2%) true seroconverter.^2^ In another single-center retrospective study of 265 psoriasis patients on TNFI’s in Cleveland, Ohio (TB burden 1.3/100,000), only 1 patient (0.4%) seroconverted.^3^ Studies conducted in countries with higher-TB prevalence reported higher seroconversion rates. A retrospective study of psoriasis patients taking TNFI’s in Taipei, Taiwan (TB burden 30/100,000), reported 7.3% of patients seroconverting.^4^ In a retrospective study psoriasis patients taking TNFI’s in Ankara, Turkey (TB burden 16/100,000), 5.2% seroconverted.^5^

Limitations include small sample size, a single-center setting, and a retrospective design.

In sum, we found a low rate of seroconversion (<1%) in patient taking biologics for psoriasis and hidradenitis suppurativa in NYC, which has relatively high TB burden. True seroconverters were older, on average, than other subjects. We recommend a single baseline QFT test and annual testing only for patients age ≥66 taking biologics for dermatologic conditions, which may reduce costs and avoid unnecessary treatments.

## Conflicts of interest

Dr Lipner has served as a consultant for Ortho-Dermatologics, Moberg Pharmaceuticals, Eli Lilly, and BelleTorus Corporation. Authors Katsiaunis, Conway, and D'Angelo have no conflicts of interest to declare.

## References

[1] (2022). State & Local Data.

[2] Ya J., Khanna U., Havele S., Fernandez A.P. (2020). Utility of repeat latent tuberculosis testing with the QuantiFERON-TB Gold test in patients with psoriasis treated with tumour necrosis factor-α inhibitors at a single U.S. institution. Br J Dermatol.

[3] Chung J., Aronson A.B., Srikantha R., Vogelgesang S.A., Wanat K.A. (2018). Low conversion rate of QuantiFERON-TB Gold screening tests in patients treated with tumor necrosis factor inhibitors: a retrospective cohort study identifying an important practice gap. J Am Acad Dermatol.

[4] Hsiao C.Y., Chiu H.Y., Wang T.S., Tsai T.F. (2017). Serial QuantiFERON-TB Gold testing in patients with psoriasis treated with ustekinumab. PLoS One.

[5] Akdogan N., Dogan S., Gulseren D. (2021). Serial Quantiferon-TB Gold test results in 279 patients with psoriasis receiving biologic therapy. Dermatol Ther.

